# Developing a knowledge translation program for health practitioners: Allied Health Translating Research into Practice

**DOI:** 10.3389/frhs.2023.1103997

**Published:** 2023-02-17

**Authors:** Adrienne M. Young, Ashley Cameron, Nina Meloncelli, Sally E. Barrimore, Katrina Campbell, Shelley Wilkinson, Liza-Jane McBride, Rhiannon Barnes, Sally Bennett, Gillian Harvey, Ingrid Hickman

**Affiliations:** ^1^ Dietetics and Food Services, Royal Brisbane and Women’s Hospital, Brisbane, QLD, Australia; ^2^ Centre for Health Services Research, The University of Queensland, Brisbane, QLD, Australia; ^3^ Office of the Chief Allied Health Officer, Queensland Health, Brisbane, QLD, Australia; ^4^ Allied Health, Metro North Health, Brisbane, QLD, Australia; ^5^ Nutrition and Dietetics, The Prince Charles Hospital, Brisbane, QLD, Australia; ^6^ Healthcare Excellence and Innovation, Metro North Health, Brisbane, QLD, Australia; ^7^ School of Human Movements and Nutrition Science, The University of Queensland, Brisbane, QLD, Australia; ^8^ School of Health and Rehabilitation Sciences, The University of Queensland, Brisbane, QLD, Australia; ^9^ College of Nursing and Health Sciences, Flinders University, Adelaide, SA, Australia; ^10^ Australian Centre for Health Service Innovation, Queensland University of Queensland, Brisbane, QLD, Australia; ^11^ Nutrition and Dietetics, Princess Alexandra Hospital, Brisbane, QLD, Australia; ^12^ Faculty of Medicine, The University of Queensland, Brisbane, QLD, Australia

**Keywords:** knowledge translation, capacity building, health workforce, allied health occupations, implementation science, mentoring, education

## Abstract

**Background:**

Front-line health practitioners lack confidence in knowledge translation, yet they are often required to undertake projects to bridge the knowledge-practice gap. There are few initiatives focused on building the capacity of the health practitioner workforce to undertake knowledge translation, with most programs focusing on developing the skills of researchers. This paper reports the development and evaluation of a knowledge translation capacity building program for allied health practitioners located over geographically dispersed locations in Queensland, Australia.

**Methods:**

Allied Health Translating Research into Practice (AH-TRIP) was developed over five years with consideration of theory, research evidence and local needs assessment. AH-TRIP includes five components: training and education; support and networks (including champions and mentoring); showcase and recognition; TRIP projects and implementation; evaluation. The RE-AIM framework (Reach, Effectiveness, Adoption, Implementation Maintenance) guided the evaluation plan, with this paper reporting on the reach (number, discipline, geographical location), adoption by health services, and participant satisfaction between 2019 and 2021.

**Results:**

A total of 986 allied health practitioners participated in at least one component of AH-TRIP, with a quarter of participants located in regional areas of Queensland. Online training materials received an average of 944 unique page views each month. A total of 148 allied health practitioners have received mentoring to undertake their project, including a range of allied health disciplines and clinical areas. Very high satisfaction was reported by those receiving mentoring and attending the annual showcase event. Nine of sixteen public hospital and health service districts have adopted AH-TRIP.

**Conclusion:**

AH-TRIP is a low-cost knowledge translation capacity building initiative which can be delivered at scale to support allied health practitioners across geographically dispersed locations. Higher adoption in metropolitan areas suggests that further investment and targeted strategies are needed to reach health practitioners working in regional areas. Future evaluation should focus on exploring the impact on individual participants and the health service.

## Introduction

The knowledge-practice gap in healthcare is substantial, with failure to optimally use evidence resulting in research waste, provision of unnecessary or potentially harmful treatments, and ineffective use of finite health service resources (1, 2). Paradoxically, a parallel knowledge-practice gap has been identified. That is, the gap between scientific knowledge on implementation (i.e., implementation science) and the use of this knowledge to implement healthcare improvements in practice (i.e., knowledge translation (KT)) (3). Simply disseminating research findings in journals or at conferences is insufficient to produce an effective or lasting change in healthcare practice. In response, implementation science has become a rapidly developing field of research, with over 100 theories, models and frameworks developed over the past two decades (4). So rife are these theoretical approaches that tools and “how-to guides” have recently been developed to help those new to the field navigate the complex language and concepts (5, 6). It has recently been suggested that these guides have too often been developed by researchers for researchers and raised questions about whether implementation science actually ever reaches healthcare practitioners so that they can use this knowledge to solve complex healthcare problems and improve outcomes (3).

The need to train researchers in dissemination and implementation research has been acknowledged for some time (7), with a recent scoping review identifying 41 distinct capability building initiatives for dissemination and implementation research (8). However, few focus on developing KT capacity within the healthcare workforce. Health practitioners are often charged with undertaking projects to translate research into practice which, given the complexity of health systems, often requires multiple non-linear steps and complex process and behavior changes of health practitioners, consumers, and decision makers (9). Given this, it should not come as a surprise that current evidence suggests health practitioners do not feel confident in their skills and ability to undertake KT, particularly when applying implementation theory, models and frameworks and evaluating the impact of the change (10–12). Without adequate training, support and resources, there is a risk of implementation failure and a sense of nihilism about KT within the healthcare workforce (13).

Developing capacity for KT has been defined as a dynamic activity that develops individual and organizational capabilities over the long term, leading to improved implementation and provision of evidence-based healthcare (14). It requires the healthcare workforce to be active participants rather than passive recipients (i.e., learning by doing, rather than by completing training courses) and a focus on multilevel learning from individuals to groups and between groups across an organization (14). Published literature on developing capacity for KT amongst the healthcare workforce has found improved knowledge and confidence, reported application of new skills, and improved local leadership and capacity for KT (15–20). These KT capacity building initiatives primarily used formal face-to-face training seminars or workshops (16, 20, 21), sometimes in combination with mentoring or coaching from “implementation support practitioners” (15, 17–19). There is emerging recognition of implementation support practitioner roles in developing KT capacity (22); however, in health organizations, they are rarely funded and, where they do exist, there may be inequitable access to their support, particularly in geographically dispersed health services.

The Queensland public health system is funded, managed, and regulated by the federal and state governments to deliver services across acute, sub-acute and community health settings, spanning an area of more than 1.8 million km2 and servicing a population of around 5.2 million people (23). Developing capacity for KT in the allied health workforce within the Queensland public health system needs to ensure equity of access to KT training and implementation support practitioners (mostly located within metropolitan centers) and delivery of these strategies in a way that fits within constraints commonly experienced in health services (e.g., time, high clinical workload, competing demands (10, 12)). In response, we developed the multimodal Allied Health Translating Research into Practice (AH-TRIP) initiative to build capacity for KT (at an individual, group and organizational level) amongst the allied health workforce working in the public health system in Queensland, Australia. Allied health includes health practitioners from non-medical and non-nursing disciplines such as dietitians/nutritionists, occupational therapists, pharmacists, physiotherapists, psychologists, medical radiation therapists, social workers, and speech pathologists.

In this paper, we describe the AH-TRIP initiative to share our approach for developing KT capacity amongst front-line allied health practitioners for other health services to learn from and build on. Specifically, this includes how AH-TRIP was developed, the inputs (resources) associated with delivery, and the evaluation of reach, adoption, and reaction of the program.

## Materials and methods

This paper uses the Medical Research Council framework for developing complex interventions (24) to describe the approach taken to develop the AH-TRIP initiative, a KT capacity building program for front-line allied health practitioners. As this framework was only recently developed, it was not applied prospectively to guide the development but provides a useful structure to describe the steps taken to develop AH-TRIP. The Template for Intervention Description and Replication (TIDieR) checklist (25) has been used to describe the AH-TRIP initiative.

A description of the AH-TRIP initiative is provided in Table 1. AH-TRIP was developed over a five-year period (2014 to 2019) and implemented across all 16 Queensland Hospital and Health Service districts, as well as a public/private partnership health service, from 2018. The development process is outlined in Table 2. Whilst this table suggests that the development process was somewhat linear, many steps were completed concurrently and/or repeated to refine the final intervention. On reflection, a pragmatic approach to intervention development was used, including approaches consistent with our values of partnership (with end users involved in working groups and steering committees to inform decision making), theory and evidence (with literature review and needs assessment at commencement informing a competency framework and development of individual intervention components), and implementation-focused (with monitoring of uptake and delivery to inform refinements) (24).

**Table 1:** Summary of the allied health translating research into practice (AH-TRIP) initiative.

AH-TRIP Components	Central Elements	Description
Training and education	Completion of key webinars (26): – Why is TRIP important? – What is TRIP? – Problems in practice: identifying an evidence-practice gap – Additional webinars related to each step in the KT process	Online repository of more than 30 webinars, 12 real-world case examples, 15 implementation science papers (selected for their accessibility to a non-academic audience) and 16 web links (26). These are promoted to end-users by AH-TRIP champions who receive weekly emails promoting selected webinars, latest research, external training opportunities etc. Content is accessed by individuals and in groups; webinars are viewed in bite-size chunks (e.g. one per staff meeting) as well as part of a longer session (e.g. one hour in-service, half-day workshop).
Support and networks	Availability of mentors with implementation expertise to provide project support to those undertaking TRIP	Statewide telementoring series (27): one-hour online group sessions for ten months to support health practitioner-led TRIP projects (up to six projects per series). The monthly sessions include an independent facilitator and an expert panel (comprised of four TRIP enthusiasts and health services leaders) who provide constructive critique and knowledge translation support. Individual project support may also be provided locally by a research fellow or workforce development officer.
	At least one nominated AH-TRIP champion per participating department/ service to promote, advocate and contribute to embedding AH-TRIP within local teams	Centralized champion network acts as a conduit between their local site and the statewide initiative. Champions receive weekly email updates to enable them to actively engage their peers in AH-TRIP in a range of ways, e.g., disseminating email updates, organizing local training sessions, promoting a TRIP approach to improvement projects, connecting individuals/teams with peer/expert support, nominating individuals/teams for local recognition and awards.
Showcase and recognition	Mechanism to recognize and celebrate individuals and teams who undertake a TRIP project and/or support capacity building within their team	Annual statewide AH-TRIP Showcase recognizes and celebrates health practitioners who have undertaken a TRIP project and acknowledges those who enable and support TRIP. The showcase provides an opportunity for health practitioners to present their TRIP projects to an audience (face-to-face and virtual) of peers and compete for a range of awards. It provides an opportunity for health practitioners in the audience and fellow presenters to learn about TRIP projects in other health services, identify and foster collaborations and consolidate TRIP knowledge. It is designed to share real-world learning about success and failure as well as reward excellence.
TRIP projects and implementation	Application of AH-TRIP principles to complete a project that meets the agreed definition of TRIP, i.e. where there is clear evidence of a local problem, i.e. a practice gap, and evidence	A variety of strategies have been used to support the completion of TRIP projects in local settings including: – Setting expectations that all projects require documented problem definition and literature review before approval to proceed with implementation – Setting targets, e.g., at least one TRIP project per team/department each year – Provide dedicated internal funding/offline time specifically for TRIP projects – Integrating AH-TRIP terminology and resources within project and quality improvement reporting templates and processes
Evaluation	Evaluation of short, medium and/or long-term outcomes of AH-TRIP initiative, consistent with program logic model	Comprehensive evaluation plan guided by a logic model (Figure 1) to evaluate the reach, effectiveness, adoption, implementation, and maintenance of the AH-TRIP initiative as a whole and individual components.

**Table 2:** Description of the steps and activities completed to develop the allied health translating research into practice (AH-TRIP) initiative.

Steps	Activities
1. Plan the development process (2014-2019)	Knowledge translation (KT) training and mentoring implemented in one metropolitan hospital dietetics department in 2014 to support critical appraisal and practice change was expanded in 2015-16 to four more metropolitan dietetics departments with internal research fellow positions. Attempts at scale and spread to other hospitals were limited by a lack of research fellow capacity across services. An alternative model to support geographically dispersed health services with limited research fellow capacity and testing in other allied health disciplines was needed. Funding secured from health organizations to appoint two project officers (total of 1.3 full-time equivalent) (2017-2019)
2. Involve stakeholders throughout the development process (2017-2019)	Development of communication and engagement plan, outlining key messages and communications tactics tailored to key stakeholders to ensure early and regular involvement. End users (i.e. allied health practitioners and managers) engaged through: – Needs assessment surveys (10, 12) – Evaluation interviews and surveys – Development of a champion network that received fortnightly email updates on new online content, invited to provide feedback, and given sharable promotional materials – Attendance at the annual showcase event – Leveraging established formal and informal statewide allied health networks, relationships between program developers Other stakeholders (research fellows, workforce development officers, university partners, allied health managers, and funders) were engaged through: – Membership on steering committees or working groups – Presentations at key meetings – Regular emails
3. Bring together a team and establish decision-making processes (2017)	Formation of a statewide steering committee to provide governance, leadership, strategic direction, and program sustainability, chaired by the Chief Allied Health Officer. Four working groups were formed, reporting to the steering committee, also with a deliberately diverse membership across allied health disciplines and geographical locations (metropolitan vs. regional): – Training and education – Support and networks – Showcase and recognition – Evaluation
4. Review published research evidence (2017)	Review of published and grey literature to identify relevant existing programs and/or strategies to develop capacity for KT; specifically, – Work by Moore, Park and Straus (18, 19) to identify content, KT competencies and evaluation measures – Identified models of telementoring (28) to ensure equity of access to “implementation support practitioners” across geographically dispersed locations.
5. Draw on existing theories (2017)	Review of implementation and evaluation theories, models, and frameworks to identify those relevant to informing the design of individual AH-TRIP components: – Knowledge to Action framework (29) used as the recommended model for undertaking knowledge translation – The RE-AIM framework (30) underpins the evaluation plan (Figure 1)
6. Articulate programme theory (2017-2019)	A logic model was developed and refined over time to articulate the elements within the AH-TRIP initiative, their inputs, activities and intended outputs and outcomes (Figure 1).
7. Undertake primary data collection (2018)	Survey of target groups to establish baseline KT awareness and self-efficacy, using a locally developed survey incorporating self-efficacy questions previously used by Park et al. (19). Respondents (*n* = 498) reported moderate confidence in identifying an evidence-practice gap, finding relevant literature/ evidence, and sharing evidence with colleagues, but low confidence in planning for and implementing change and supporting others to undertake KT (10, 12).
8. Understand context (2017-2019)	These surveys identified barriers and enablers to developing capacity for KT from the perspectives of the allied health workforce, with key findings being: – Enablers: high interest in learning more about KT – Barriers: lack of management support, lack of quarantined time, cost, and travel requirements to attend training. Diverse membership on the steering committee and working groups facilitated an understanding of context at statewide levels. Understanding of the local context was obtained through local needs assessment surveys to tailor implementation to the local context, a strong organizational commitment to research and improvement and dedicated resources to workforce development. This was enabled through local leaders and in some cases, the formation of a local AH-TRIP steering committee.
9. Pay attention to future implementation of the intervention in the real world (2017-2019)	Early and regular attention was paid to enhancing the “implementability” of AH-TRIP across settings and disciplines – Alignment with other initiatives in the health service – Inclusion in the 10-year statewide research plan – Inclusion in local hospital strategic plans – Formal sustainability assessment (31) Threat to fidelity with spread to new settings was also identified early as a potential risk, leading to the development of a toolkit to articulate central elements of the program.
10. Design and refine the intervention (2017-2019)	The AH-TRIP initiative is described in Table 1, and was designed by the working groups outlined in Step 4. The program components have been revised as each element has been trialed and evaluated and participant feedback incorporated. For example, – Training and Education: length of webinars reduced; content revised based on alignment with newly developed KT competencies (18). – Support and networks: telementoring program and face-to-face peer support groups extended from six to ten months. – Showcase and recognition: format modified to include a guest speaker (KT expert or health service leader enabling KT), additional award categories, and hybrid virtual capability added.
11. End the development phase (2019- ongoing)	There is no plan to end the development of AH-TRIP, with ‘program adaptability’ identified as a key strength for sustainability. A working group has been developed to specifically focus on program sustainability, with a formal sustainability assessment (31) conducted to identify and quantify areas for attention. In addition, financial sustainability has been calculated to sustain the status quo vs. growth (i.e., the expansion of AH-TRIP to more hospitals and health services each year).

AH-TRIP, Allied Health Translating Research into Practice; KT, knowledge translation; RE-AIM, reach, effectiveness, adoption, implementation, maintenance.

**Figure 1:**
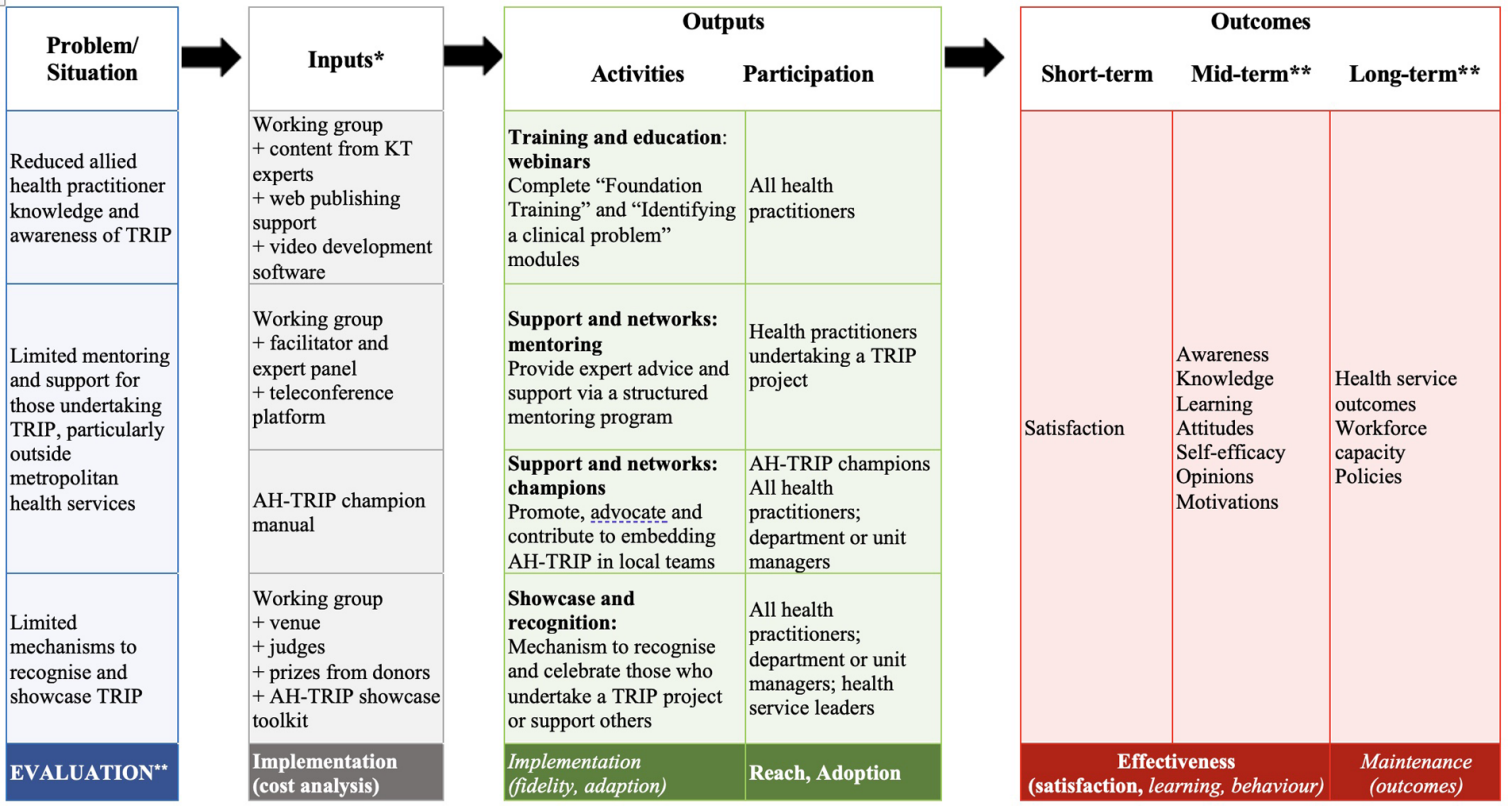
Allied health translating research into practice (AH-TRIP) program logic and evaluation. *Funded program lead positions [total 1.2 full-time equivalent (FTE) across two positions] and in-kind research fellow time (approximately 0.1FTE) were inputs for all components of AH-TRIP. **As per the RE-AIM framework(26); italicised evaluation measures are not reported in this paper.

This paper reports the cost associated with developing and delivering AH-TRIP (inputs) and the evaluation of the participation (reach and adoption) and short-term outcomes (satisfaction) over a three-year period (2019–2021). This evaluation is informed by the program logic model and underpinned by the RE-AIM (Reach, Adoption, Effectiveness, Implementation, Maintenance) framework (30) (Figure 1). Implementation and learning outcomes of the telementoring program have been reported elsewhere (27, 32).

Inputs are defined as the resources involved in delivering AH-TRIP, including funding, time and materials (33); these are outlined in detail in Figure 1. Data were collected *via* documents from the AH-TRIP program manager and working groups (e.g., meeting minutes) to estimate the funded and in-kind time (i.e., not funded) dedicated to AH-TRIP by all contributors. Cost of materials were calculated *via* invoices (for purchased materials, e.g., software licenses) and estimates for those donated or provided in-kind (e.g., prizes, venue hire). All costs are reported in Australian dollars.

Reach was defined as the number and representativeness of individuals who participated in the AH-TRIP telementoring, champion and showcase initiatives in 2019, 2020 and 2021, and the number of page views for the webinars between January 2019 and December 2021. The AH-TRIP program manager collected participation data *via* attendance lists (telementoring and showcase), submissions to present a TRIP project (showcase), and email list (champions). Website statistics were obtained from the health service publishing team. Adoption was defined as the number of hospital and health service districts with at least one AH-TRIP champion and at least one TRIP project supported by AH-TRIP (determined by either submission to the telementoring program or showcase).

Participant satisfaction with the AH-TRIP components was determined using surveys administered after completion of AH-TRIP webinars (available as a link on the webpage) and after participation in AH-TRIP telementoring and showcase (provided to all participants directly *via* email/QR code). Copies of these surveys are provided as Supplementary Material. Approval to undertake this program evaluation was granted by the chairperson of the hospital human research ethics committee (LNR/2019/QRBW/57225). Participants gave their consent to participate in the evaluation by their completion and return of their surveys.

All data are presented descriptively for the AH-TRIP initiative in its entirety, as well as evaluation for each individual component. Where possible, reach and adoption are also reported by geographical location (metropolitan vs. regional hospitals and health services) and allied health discipline to determine the representativeness of participating individuals and sites.

## Results

The cost to deliver the AH-TRIP initiative was $AU197,595 per year. This includes $AU143,000 for two funded positions for a total of 44 h per week: 0.6FTE statewide program manager to support the statewide coordination and delivery of all AH-TRIP components; 0.5FTE implementation practitioner funded internally by a specific metropolitan health service to support local mentoring and contribute to the delivery of select statewide AH-TRIP components. Additional costs included $AU475 in software licenses, and direct in-kind contribution of $AU54,120 per year (e.g., steering committee and working group meetings, webinar content development, telementoring panelists, showcase event expenses). Detailed cost data and assumptions are provided as Supplementary Material.

**Figure 2:**
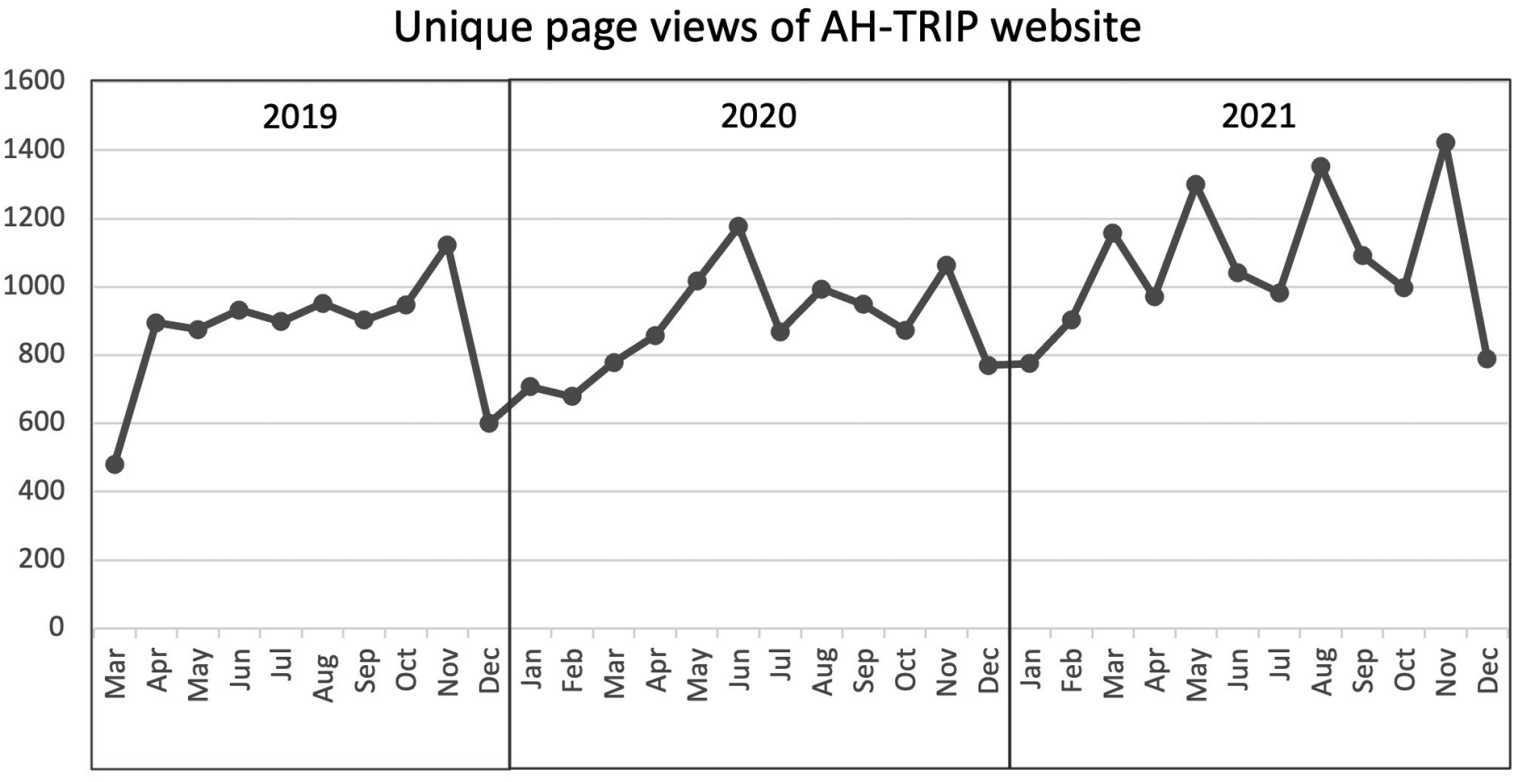
Unique page views of the allied health translating research into practice website.

The AH-TRIP website that hosts online training and resources was launched in March 2019. Between its launch in March 2019 and December 2021, there was an average of 944 unique page views per month (range: 480–1,422 per month), with total unique page views of 32,112 over this period (Figure 2). Only 19 surveys were completed online to evaluate the satisfaction with these resources. The AH-TRIP champion network comprised of more than 100 champions (2019: *n* = 103, 2020: *n* = 112, 2021: *n* = 105) who promoted AH-TRIP and knowledge translation within their departments. The champions represented all major allied health disciplines (Table 3), with a third of champions (*n* = 36) located in regional health services.

**Table 3:** Project topics supported by telementoring or presented at the showcase.

	Telementoring support	Showcase presentation
Implementing and/or evaluating evidence-based models of care	– Falls prevention – Family involvement in care – Gastrostomy management – Healthy weight gain during pregnancy – Home-based exercise after hip surgery – Inpatient dementia management – Inpatient eating disorders – Inpatient nutrition and mobility – Nutrition in chronic kidney disease – Occupational therapy for chronic disease – Outpatient diabetes management – Peri-operative nutrition and mobility interventions – Telehealth services – Student-led psychology clinics	– Inpatient nutrition and mobility – Paediatric feeding clinic within remote health service – Peri-operative nutrition and mobility interventions – Pre-admission social work service – Weight management outpatient services – Staff education and family involvement for disorders of consciousness – Inpatient eating disorders – Phenylketonuria management – Breast cancer lymphoedema – Peri-natal education – Telehealth services
Understanding barriers and/or implementing screening/ assessment processes	– Cognitive assessment – Dysphagia screening	– Body composition measurement – Colorectal cancer screening – Measurement of core outcomes – Sarcopenia and frailty assessments

The initiation of the AH-TRIP telementoring program supported 19 knowledge translation projects across four cohorts (2019: *n* = 4, 2020: *n* = 5, 2021: *n* = 10 across two cohorts). Project topics are summarized in Table 3. Twenty-five allied health practitioners across seven disciplines and one nurse participated in the program as “telementees” (Table 4). Seven (37%) projects were led by allied health practitioners from regional health services (2019: *n* = 2; 2020: *n* = 1; 2201: *n* = 4 projects across two cohorts), with a total of nine individuals from regional health services supported by the program. All “telementees” (100%, *n* = 26) reported that the telementoring support they received was relevant to their project, and all (100%, *n* = 26) reported that they would recommend it to their colleagues. Local program funding from one metropolitan health service district enabled individual project support by an experienced implementation practitioner for an additional 99 projects involving allied health practitioners.

**Table 4:** Number of allied health practitioners participating in each allied health translating research into practice (AH-TRIP) program component (2019–2021) by allied health discipline.

AH-TRIP program component	Dietetics	Occupational therapy	Pharmacy	Physiotherapy	Psychology	Radiation therapy	Social work	Speech pathology	Other^a^
Champions (*n* = 105)^b^	29	23	3	19	3	2	5	16	5
Group telementoring (*n* = 26)	7	6	1	6	2	0	2	2	0
Showcase: submissions (*n* = 49)	23	5	2	9	0	0	3	5	2

^a^ Includes: exercise physiology, audiology, clinical measurements, podiatry, radiography, oral therapy, and art therapy.

^b^ This data represents champions from 2021.

Completed TRIP projects were submitted for the AH-TRIP annual showcase event by 49 allied health practitioners (2019: *n* = 17, 2020: *n* = 19, 2021: *n* = 13). Almost half of these (*n* = 23) were submitted by dietetics (Table 4), with representation from both metropolitan (*n* = 38) and regional areas (*n* = 11). Project topics presented at the showcase are summarized in Table 3. A total of 733 people registered to attend the showcase events over the evaluation period (2019: *n* = 285, 2020: *n* = 263, 2021: *n* = 185). This included 186 (25%) attendees from regional areas of Queensland and 89 (12%) from outside Queensland Health (e.g., universities, private practice). Evaluation surveys were completed by 146 attendees (2019: *n* = 76, response rate: 27%; 2020: *n* = 43, response rate: 16%, 2021: *n* = 27, response rate: 15%). Almost all respondents agreed that the event was valuable (*n* = 138, 95%), could identify at least one key learning about TRIP from the event (*n* = 138, 95%), would think about how they used research in practice because of the event (*n* = 140, 96%) and had increased understanding and confidence about what TRIP is (*n* = 126, 86%). Qualitative comments showed that attendees particularly liked that the showcase provided a safe space to share reflections and learning, including failed TRIP attempts and “real world learnings” from across the state, and that it was an engaging way to learn about research and TRIP.

Over the three-year evaluation period, nine out of the 16 Queensland Hospital and Health Service districts, as well as the private/public health service, adopted AH-TRIP, with all ten having at least one AH-TRIP champion, four having at least one AH-TRIP project supported by the telementoring program and nine having submitted a project to the annual showcase event. Of those seven hospital and health service districts who had not fully adopted AH-TRIP, six had partially adopted AH-TRIP (at least one AH-TRIP champion, *n* = 4; project supported by the telementoring program, *n* = 1; submitted to present at the showcase, *n* = 1). All non or partial adopters were located in regional areas.

## Discussion

In this paper, we have shared both the development and evaluation of this multimodal KT capacity building program that has resulted in strong adoption and high participation within its first three years of implementation. Almost 1,000 allied health practitioners have participated in AH-TRIP through being a local champion, receiving mentoring, or attending the showcase. The online training platform has received over 1,000 unique page views each month, and over 60 known TRIP projects have been undertaken across the state. This has been achieved even during times of high demands on health services and health practitioners due to the COVID-19 pandemic. Importantly, evaluation demonstrates some penetration in health services outside of metropolitan centers, working towards achievement of its aim to provide equitable access to KT capacity building for health practitioners in regional areas who typically do not access the same support as their colleagues in metropolitan areas. AH-TRIP fills an identified gap in KT capacity building by specifically focusing on novice implementers and teams embedded in the health service rather than advancing dissemination and implementation skills of individual researchers (8).

The AH-TRIP initiative was developed with five key components: training and education; support and networks (including mentoring); showcase and recognition; TRIP projects and implementation; and evaluation. Although we provide a summary of the steps and activities to developing the initiative to date, this non-linear process has required continuous monitoring and adaptation to achieve the scale and spread across a geographically large health service with a workforce of over 9,000 allied health practitioners. Critical success factors of the AH-TRIP pilot sites were identified as the collaborative engagement, teaching and coaching by research fellows (acting as “implementation support practitioners” (22)), operational support from key opinion leader managers, a motivated workforce with an expressed desire for TRIP training (10, 12) and recognition of effort at an annual showcase. The formation of formal governance structures such as a steering committee and working groups with clear reporting lines were crucial to securing ongoing, albeit short term funding for project officer support. Local needs assessment surveys influenced success by generating data by which decisions to support or invest in local AH-TRIP activities or projects were made. Consistent with findings from previous studies (16, 20), a strong organizational commitment to research and quality improvement and dedicated resources to workforce development were clearly identified as factors associated with the sites that were able to integrate AH-TRIP successfully.

Attempts at scale and spread to other hospitals were limited by research fellow capacity in regional health services (34) and the uncertainty of the short-term, non-recurring nature of funding new program initiatives within health services during the initial years of AH-TRIP establishment. The lack of physical location of research fellows at each regional location was a challenge that was not overcome simply with virtual engagement. The awareness and recognition of implementation science and KT as distinct teachable skills for a healthcare workforce is still evolving within health services. There remains some permeation of expectation across levels of health service management that staff should be able to identify evidence practice gaps and implement, evaluate, and sustain knowledge translation without dedicated training and support to do so. The AH-TRIP experience has demonstrated that with a relatively small investment, the rewards of KT training are broad across disciplines and project types.

Whilst this and other studies (27, 32) have demonstrated high engagement and KT skill development of allied health practitioners participating in AH-TRIP, measuring its value is challenging. Since its inception, considerable “in-kind” time has been provided by research fellows, allied health practitioners and managers to develop, implement and sustain AH-TRIP, which is not possible to quantify. However, based on the accounted costs, the cost of the initiative is $200 per participant or $19,760 per year for each hospital and health service district that has adopted AH-TRIP. This is likely to be far less expensive than more traditional models of education and mentoring requiring dedicated positions within each hospital and health service district. To measure the true value of AH-TRIP, there is a need to capture the impact beyond short-term skill development and project completion, to measure impact on the health system and patient outcomes. Data is also needed to demonstrate the impact of capacity building for the individual and potential “ripple effects” as capabilities are spread within groups and organizations (14) (e.g., mentoring of others in KT, integration of KT into policies and processes). It is possible that enhancing KT capacity of health service staff may provide them with a new language, skills and networks to forge new academic partnerships, providing the opportunity to undertake larger-scale funded KT projects within a research framework. An important consideration when measuring the value of KT capacity building is capturing the cost associated with the avoidance of poorly executed projects. It is our experience that AH-TRIP mentoring supports health practitioners to take a slower and more structured approach to problem definition and selection of implementation strategies (27); however, to our knowledge, this has not been evaluated to date.

Participation in AH-TRIP by allied health practitioners in regional areas demonstrates that it is suitable to be delivered at scale and can overcome barriers related to geographical distance, congruent with the aims of the initiative. However, the lack of adoption in seven regional hospital and health service districts suggests that further work is needed to enable adoption by the health service and participation by the workforce. Slower adoption in regional, rural, and remote health services was expected, with previous research highlighting lower levels of prior KT training outside metropolitan health services (10). In response, we have realigned our working groups to have one specifically focused on regional workforce engagement, with strategies such as positions in the AH-TRIP steering committee and working groups filled by regionally located practitioners/champions, dedicated rural and remote bursaries to support AH-TRIP activities and practical support specifically offered to regional projects by the program manager, e.g., review and feedback on resources, project materials and showcase submissions. Three years on, there continues to be a need for ongoing resourcing to refine and sustain AH-TRIP and adaption of the delivery approach and content within the initiative with advancement in implementation science knowledge and capacity building theory, as well as inevitable changes in the health service practice setting and ecological system (35).

Some limitations should be noted. Without dedicated evaluation funding, data used in this evaluation was limited to those embedded within routine delivery of the program related to reach, adoption, and satisfaction. These data were not available for users of the open-access online training resources on the AH-TRIP website; with fewer than twenty surveys completed over three years, alternative strategies should be used to evaluate the online training. Evaluation of any additional KT training and mentoring provided by AH-TRIP champions and research fellows beyond the core components was not undertaken. It is likely that the cost of AH-TRIP has been underestimated due to significant in-kind contributions from a wide range of supporters who engaged indirectly in AH-TRIP development and promotion that was unable to be quantified or costed. Whilst the authors were also heavily involved in the development and delivery of the AH-TRIP program, inclusion of two external evaluators with expertise in KT capacity building (SB, GH) may have reduced bias in interpretation of evaluation data. Finally, we acknowledge that our definition of adoption represents a minimum level of adoption of the program and does not capture whether it has been adopted and embedded within multiple discipline groups or individual health care facilities within the hospital and health service district.

In conclusion, AH-TRIP is a low-cost KT capacity building initiative which can be delivered at scale to support allied health practitioners across geographically dispersed locations. Specific strategies are needed to further support the adoption of AH-TRIP in regional health services, and to ensure its sustainability within health services that have adopted the initiative. Future evaluation should focus on exploring the impact on individual participants, their teams, and organizations, as well as the potential impact on health service outcomes.

## Data Availability

The original contributions presented in the study are included in the article/**Supplementary Material**, further inquiries can be directed to the corresponding author/s.
