# Supplementary material for: Developing a knowledge translation program for health practitioners: Allied Health Translating Research into Practice
Source: Front Health Serv. 2023 Feb 17;3:1103997. doi: 10.3389/frhs.2023.1103997 (PMC10012769; doi:10.3389/frhs.2023.1103997)
Supplement: Supplementary file 1 [file Table1.docx]

Supplementary Material

# Evaluation surveys

## AH-TRIP telementoring surveys:

Online survey administered to participants at the completion of the program

| Evaluation domain | Question | Response options |
| --- | --- | --- |
| Implementation barriers | Over the course of the AH-TRIP Support Sessions, was your access to technology adequate for your needs? | Yes/No |
| Implementation barriers | How much time on average per month did you spend on activities related to the AH-TRIP Support Sessions? | Free text |
| Reaction | Was the AH-TRIP support you received relevant to your practice? | Not at all/ No/ Maybe/ Yes/ Very relevant |
| Effectiveness | Please rate your confidence in your ability to undertake each of the stages in a TRIP project:   - I can conduct a needs assessment for health practitioners to undertake evidence-based practice - I can adapt research evidence to the needs of local health practitioners/ stakeholders (e.g. clinicians, healthcare managers) - I can identify barriers and facilitators to knowledge uptake across different health practitioners - I can develop an evidence-based knowledge translation (TRIP) intervention based on assessment of barriers and facilitators to knowledge uptake - I can develop a strategy for monitoring knowledge use - I can develop a strategy for evaluating relevant outcomes from knowledge use - I can develop a strategy for sustaining knowledge use over time | For each statement:  Terrible/ Very poor/ Poor/ Not sure/ Good/ Very Good/ Excellent |
| Effectiveness | What has been your greatest learning? | Free text |
| Maintenance | How are you going to continue to build your TRIP skills and confidence now the support sessions have ended? |  |
| Effectiveness | Your involvement in the AH-TRIP initiative has lead to...   - Finding likeminded people - Thinking more about my project and making subsequent amendments - Consulting and/or recruiting people to support me and my project (e.g. new investigator, support, mentor, contributor from work area) - Becoming a TRIP leader or support person - Initiating TRIP activity, support and/or advice - Recommending others engage with AH-TRIP - Recommending others use a TRIP approach for relevant projects - Being more likely to use a TRIP approach for future projects - Other (please specify) | Checkbox (choose all that apply) |
| Maintenance | Do you plan to submit your project to the AH-TRIP Showcase event in 2021? | Yes/ Maybe/ No/ I have previously submitted to the AH-TRIP showcase/ I don’t know what the AH-TRIP showcase is |
| Reaction | Would you recommend the AH-TRIP Support Sessions to a colleague? | Definitely not/ No/ Maybe/ Yes/ Absolutely |
| General | Any other comments or feedback? | Free text |

## AH-TRIP showcase surveys:

Online survey administered to all attendees at the completion of the event

| Evaluation domain | Question | Response options |
| --- | --- | --- |
| Reach | What is your health profession? | Radio buttons |
| Reach | Which Hospital and Health Service (HHS), university or institution do you work with? | Radio buttons |
| Reaction | What has been your experience of the AH-TRIP Virtual Showcase?   - I valued the AH-TRIP Virtual Showcase - I could identify a minimum of one learning from this event - The AH-TRIP Virtual Showcase presentations made me think about how I use research in my practice - This event has increased my understanding of translating evidence into practice - I intend to access the AH-TRIP resources and/or opportunities - I would recommend attending a future AH-TRIP Virtual Showcase to friends or colleagues - I am likely to submit a TRIP project to next year's event | Strongly/ somewhat disagree/ neither agree nor disagree/ somewhat agree/ strongly agree |
| Reaction | What did you value most of the AH-TRIP Virtual Showcase? | Free text |
| Implementation | Before registering for this event were you familiar with the AH-TRIP initiative? | Yes/ No |
| Adoption | Have you accessed AH-TRIP resources or support?   - Webinars - Resources - Toolkit(s) - Telementoring Support - 1:1 Support - Pre-Showcase Support - No - Other (please specify): | Checkbox (choose all that apply) |
| Reach | Are you a member of the AH-TRIP initiative?   - AH-TRIP Steering Committee member - AH-TRIP Working Group member - AH-TRIP Champion - AH-TRIP Enthusiast - No - Other (e.g., AH-TRIP Telementoring Support) | Checkbox (choose all that apply) |
| General | Do you have any comments or suggestions for this event? | Free text |

# AH-TRIP annual costs

| AH-TRIP component | Description | Personnel | | Other |
| --- | --- | --- | --- | --- |
|  |  | **Funded** | **In-kind** |  |
| All | Statewide program manager: average 0.6 FTE per year (0.4 FTE 2019; 0.8 FTE 2020; 0.6 FTE 2021) at Advanced Health Practitioner level  (1.0FTE $130,000) | 78,000 |  |  |
| All | Metro North Health workforce development officer (AH-TRIP): 0.5 FTE per year at Advanced Health Practitioner level  (1.0FTE $130,000 per year) | 65,000 |  |  |
| All | Research fellow: 0.1 FTE at Advanced Health Practitioner level |  | 12,900 |  |
| All | Statewide steering committee: 12 members attending 4 meetings per year (1hr meeting plus  1 hr preparation, $100/hr) |  | 9,600 |  |
| All | Evaluation working group: 8 members attending 4 meetings per year (1hr meeting plus 1 hr preparation, $70/hr) |  | 4,480 |  |
| Training and education | Videoscribe for webinar development |  |  | 265 |
| Training and education | Education and Training working group: 8 members attending 4 meetings per year  (1hr meeting plus  1 hr preparation, $70/hr) |  | 4,480 |  |
| Training and education | KT expert time for webinar development: 5 webinars per year (2hr each, $100/hr) |  | 700 |  |
| Training and education | Clinician time for case study development: 4 case studies per year (2hr each, $60/hr) |  | 480 |  |
| Support and networks | Telementoring working group: 8 members attending 4 meetings per year (1hr meeting plus  1 hr preparation, $70/hr) |  | 4,480 |  |
| Support and networks | Panel members: 3 panel members attending 6 telementoring sessions per year  (1hr meeting plus  2 hr preparation, $100/hr) |  | 5,400 |  |
| Support and networks | Zoom |  |  | 210 |
| Showcase and recognition | Showcase working group: 8 members attending 4 meetings per year (1hr meeting plus 1 hr preparation, $70/hr) |  | 4,480 |  |
| Showcase and recognition | Judges: 3 judges, 2x hours per year ($100/hr) |  | 600 |  |
| Showcase and recognition | Venue hire: 4 hours per year |  |  | 720 |
| Showcase and recognition | Catering and prizes: |  |  | 5,800 |
| Total per year |  | **143,000** | **47,600** | **6,995** |

**Assumptions:** All meetings are one hour in duration plus one hour of preparation; telementoring is one hour with two hours preparation. No out-of-session time has been factored into working group estimates. Costs calculated based on average number of people attending meetings.  Wage estimates: steering committee members, KT experts, judges: $100 per hour; working group members: $70 per hour; clinicians: $60 per hour. Zoom and VideoScribe costs based on individual licenses; room hire based on cost for external hire.
